# Development of the Gut Microbiome in Children, and Lifetime Implications for Obesity and Cardiometabolic Disease

**DOI:** 10.3390/children5120160

**Published:** 2018-11-27

**Authors:** Anica I. Mohammadkhah, Eoin B. Simpson, Stephanie G. Patterson, Jane F. Ferguson

**Affiliations:** 1 Division of Cardiovascular Medicine, Vanderbilt University Medical Center, Nashville, TN 37232, USA; anica.i.mohammadkhah.1@vanderbilt.edu (A.I.M.); simpsone@tcd.ie (E.B.S.); 2 Division of Critical Care Medicine, Department of Pediatrics, Vanderbilt University Medical Center, Nashville, TN 37232, USA; stephanie.g.patterson@vumc.org

**Keywords:** microbiome, obesity, cardiometabolic disease

## Abstract

Emerging evidence suggests that microbiome composition and function is associated with development of obesity and metabolic disease. Microbial colonization expands rapidly following birth, and microbiome composition is particularly variable during infancy. Factors that influence the formation of the gut microbiome during infancy and childhood may have a significant impact on development of obesity and metabolic dysfunction, with life-long consequences. In this review, we examine the determinants of gut microbiome composition during infancy and childhood, and evaluate the potential impact on obesity and cardiometabolic risk.

## 1. Introduction

**Figure 1:**
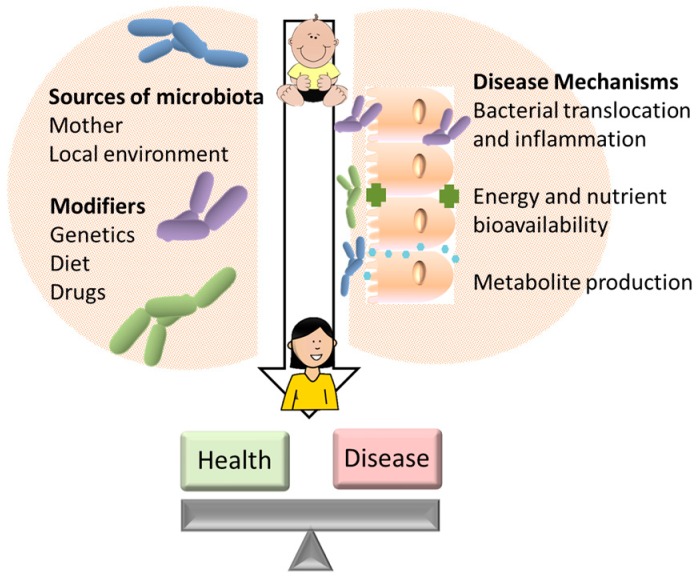
Determinants of microbiome composition, and potential mechanisms linking microbiota to disease.

Chronic cardiometabolic diseases are the leading cause of mortality in the US, with heart disease and diabetes costing >$500 billion a year [1,2], and affecting around 60% of individuals over their lifetime [1,3]. Many factors contribute to disease risk, including environmental exposures, diet, and genetic background. Pathogenesis develops over decades, making risk factors that originate in childhood of particular importance for life-long disease risk. The role of the microbiome as a disease modulator is being increasingly recognized and studied [4,5]. The human gut microbiome may act as a central regulator of metabolism, responsive to alterations in dietary intake or host physiology [6,7]. The gut microbiome expands rapidly in infants following birth [7,8] and dysbiosis in infancy or childhood may affect the long-term health of the gut microbiome. Within the sections of this review, and as summarized in Figure 1, we evaluate several of the known determinants of microbiome composition and examine how variation in microbiome composition in children may impact lifetime risk of obesity and cardiometabolic disease.

## 2. Determinants of Gut Microbiome Colonization in Neonates

### 2.1. Delivery Method

Extensive colonization of the infant microbiome occurs at the time of birth, and may even occur before birth. Inoculation has stochastic components, with contribution of maternal microbiota, but also opportunistic colonization by microbiota present on other proximal individuals and in the local environment. Gut microbiome composition is highly variable in early infancy, and of the strains that reach the infant gut, only a subset successfully colonize [7]. In a study of 25 mother–infant pairs, the development of the infant microbiome was assessed from birth to 4 months postpartum [9]. The early infant microbiome was found to contain maternal vaginal, skin, oral, and fecal strains, with variability in which site contributed the most, despite all infants being delivered vaginally. Skin and vaginal transmission appeared to be transient, with the infant gut microbiome having greatest similarity to the maternal gut microbiome by 4 months post-birth [9]. Several studies have examined the effect of vaginal delivery compared with cesarean section in order to test the hypothesis that initial exposure to vaginal vs. skin microbiota has long-term effects on microbiome composition. The intestinal colonization rate of infants delivered by Caesarean has been shown to be delayed [10], with lower bacterial colonization rates in infants delivered by cesarean section [11]. Lower microbial diversity has been observed in infants born by Caesarean section, an effect which may persist for at least two years [12]. However, in another sample, diversity was lower in preterm infants born vaginally compared with cesarean section [13], which could potentially be linked to the additional birth complications for preterm infants. Caesarean section has been associated with some long-term outcomes, including overweight [11,14,15,16], although other studies have found no differences, and causality has not been established [17,18].

### 2.2. Preterm Birth

Preterm infants have underdeveloped intestines, which may perturb the development of healthy host–microbiota relationships early in life [19]. Preterm infants are at a much higher risk of developing complications following birth, including necrotizing enterocolitis (NEC), which may be linked to impaired gut microbiome acquisition [20]. Preterm infants who developed NEC were found to have increased abundance of Proteobacteria and decreased abundance of Firmicutes and Bacteroidetes [20]. There is also evidence that altered maternal microbiome composition may be related to risk of preterm birth [21], suggesting that altered microbiome composition is both a cause and a consequence of preterm birth. Preterm infants have been shown to have differences in microbiome diversity, dependent on gestational age [22]. Dysbiosis in the preterm infant gut has been linked to delivery method, steroid use, and antibiotic use, all of which affect intestinal microbiome development [13,23,24].

### 2.3. Pre-Natal Microbial Colonization

Whether maternal transmission of commensal microbes to the fetus is a common occurrence remains an open question. Until relatively recently, the uterine environment was thought to be sterile. This assumption has been challenged by several studies finding evidence of microbiota in the uterus [25], placenta [26], umbilical cord [27], and amniotic fluid [28], as well as in meconium [29], indicating that microbiota may be transmitted during fetal development. However, data are conflicting [30,31]; microbial DNA may be detected due to contamination, presence of DNA from dead bacteria, or collection after membrane rupture [32], and thus far microbes have not been proven to be viable in utero. While evidence supports some transmission of microbial material to the fetus, whether this is a significant contributor to a fetal microbiome, and whether this has an impact on development or outcomes, remains to be determined.

## 3. Effects of Early Infant Feeding Practices on Microbiome Development

### 3.1. Breast Milk and Formula Feeding

It is thought that breast milk feeding is protective against development of multiple diseases, including obesity [33], diabetes [34], and potentially also immune-mediated diseases such as asthma and allergies [35]. The mechanism whereby breast milk determines a child’s predisposition to cardiometabolic and inflammatory disease is yet to be fully determined, but may be mediated through long-term effects on gut microbiota. In particular, as the sole source of nutrition in the first 4–6 months of life, the composition of breast milk or formula determines nutrient availability to gut microbiota in the infant, and may exert selective pressure. A key difference between breast milk and formula is the presence of prebiotics, oligosaccharides, and antibodies, which can selectively modulate bacterial abundance [36]. Breast milk itself contains microbiota, including *Bifidobacterium*, *Streptococcus*, and *Lactobacillus* species [37,38,39], which may contribute directly to the infant gastrointestinal microbiome. However, there is large variability in the composition of human breast milk [40,41,42], which is modulated by maternal health status [43,44,45]. The composition of breast milk is dynamic, changing over time, and may be responsive to infant characteristics such as sex [46], or dynamic cues during illness. Whether a “core” group of breast milk components common to most individuals are responsible for most of the protective effects remains to be determined [47]. In preterm infants, breast milk feeding appeared to mitigate some of the negative consequences of low birth weight on the development of the microbiome [48]. Studies of microbiomes in infants have focused on specific bacterial abundance, as well as diversity. In one study, breast-fed infants were found to have greater numbers of *Bifidobacterium*, but the microbiome of formula-fed infants was more diverse [49]. Another study found enrichment of Actinobacteria and Firmicutes and depletion of Proteobacteria in breast-fed compared with formula-fed infants [50]. Formula feeding has been associated with altered bacterial diversity in infants [51], with the microbiota of infants fed both breast milk and formula being more similar to formula-fed infants than to exclusively breast-fed infants [33]. Formula feeding at 3 months was associated with greater risk of overweight at 12 months, defined as infants >85th percentile for weight for length [33]. In a prospective study, children who were overweight at age 7 had lower *Bifidobacterium* and higher *Staphylococcus aureus* colonization in infancy compared with matched normal weight children [52]. Breast milk-derived immunoglobulins have been shown to modulate intestinal immune function and gut microbiome composition [53], providing further evidence for mechanisms linking breast milk feeding with immunoprotection. In a population at risk of undernutrition, lower levels of sialylated oligosaccharides in breast milk were found to be associated with stunted infant growth, and inclusion of sialylated oligosaccharides in the diet of lab animals was associated with body mass in a gut microbiome-dependent manner [54]. Although many more studies are required, these data highlight early infancy as a critical period where microbial dysbiosis may lead to overweight in later life because the microbiome may be unable to recover from dysbiosis established early in life. Components in breast milk may shape the infant microbiome to confer lifelong protection against obesity and other metabolic diseases. However, given the large variability in breast milk composition, and the potential for interaction with genetic background, there may also be cases where breast milk promotes less favorable microbiome development.

### 3.2. Prebiotic and Probiotic Supplementation

The specific composition of different types of formula may modulate the microbiome. Several trials have assessed the inclusion of probiotics or prebiotics such as oligosaccharides in infant formula to more closely mimic breast milk composition [55]. Infant formula supplemented with several *Bifidobacterium* strains altered microbiome composition in infants, but did not affect long-term colonization [56]. There was no significant effect of oligosaccharide and *Bifidobacterium* supplementation on diarrhea or febrile infection, however, the microbiota of supplemented infants more closely resembled that of breast-fed infants [57]. Inclusion of lactose in hydrolyzed formula designed for infants with milk allergies promoted growth of *Bifidobacterium* and *Lactobacillales*, and increased intestinal short chain fatty acids (SCFAs) [58]. *Lactobacillus* supplementation was found to alter gut microbiome composition [59]. Current data suggest that inclusion of pre- and probiotics in formula is well-tolerated, however, whether this has beneficial effects on longer-term outcomes is not yet known.

### 3.3. Milk Delivery Method

Some evidence exists on different effects of direct breast feeding versus providing expressed breast milk from a bottle [60]. During breast feeding, infants are exposed to maternal skin microbiota, and also deposit saliva, which contains microbiota and pathogens that can be transmitted back to the mother, potentially eliciting changes in breast milk composition through a feedback loop [61,62]. While intriguing, this area requires further research.

### 3.4. Donor Breastmilk

Because of the potential benefits of breastmilk, donor milk is sometimes used when milk from the infant’s biological mother is not available. This is particularly promoted in preterm infants. However, whether donor milk has the same protective properties remains unclear [63]. In a randomized trial in preterm infants, donor milk did not appear to confer an advantage over formula when compared with maternal milk [64]. Donor milk is general pasteurized to reduce risk of infection and is often pooled from multiple donor sources. Pasteurization may destroy pre- and probiotics, reducing the beneficial effects of human milk. Further, the variability in breast milk composition may result in donor milk being suboptimal for an unrelated infant. However, much more research is required to establish the potential benefits and risks of using donor milk as an alternative to formula.

## 4. Dietary Modulators of Gut Microbiome Composition throughout Childhood

The introduction of solid food is associated with a shift in the infant microbiome to more closely resemble adult profiles, however, the pediatric microbiome remains in flux for at least the first 3 years of life [7]. This suggests a period of relatively malleability and implies that diet in early childhood may have a disproportionately large impact on lifetime microbiome composition and associated health impacts. In adults, a change in diet significantly affects the composition of their gut microbiome, with observable major shifts in microbe composition within 24 h of substantial or acute alterations to the diet, such as suddenly switching to solely plant- or animal-based foods. A near return to the starting composition can be observed 48 h after resumption of the normal diet [6]. Data are limited on the effect of dietary intervention on the microbiome in children, but given that microbiome profiles in children after the age of 3 years closely resemble those of adults, it is likely that dietary changes can rapidly affect microbiome composition in children.

### 4.1. The Effect of Western Diet on the Gut Microbiome

The Western diet, high in animal protein and fat, high in refined carbohydrates, and low in plant-derived fiber, phytochemicals, and fermented foods has been associated with the relatively recent rapid rise in inflammatory-related diseases, including cardiometabolic and intestinal disease. There is increasing evidence that this may be mediated through gut microbiota [65,66]. The Western diet has been found to decrease total bacterial load, as well as those of beneficial genera such as *Bifidobacterium* and *Eubacterium* [67]. Studies of vegetarian and vegan diets have found varying levels of differences in microbiome composition compared with the typical unrestricted Western diet, with potential negligible differences in the overall functional capacity of the microbiota [68,69,70,71]. A study investigating the impact of the Western and traditional plant-based diet in children in Thailand found differences in composition and used metagenomic prediction to identify underrepresentation in genes for butyrate biosynthetic pathways in children consuming a Western diet [72], which may modulate gut immune homeostasis [73]. Similarly, children consuming traditional diets in rural Malawi and Venezuela were found to have differences in metabolic gene content of the microbiome compared with US children consuming a Western diet [7]. In a small study of urban visitors to a traditional rainforest village, the microbiome of children was found to be more prone to change compared with a relatively stable microbiome in adults, highlighting the higher plasticity of the microbiome in children [74]. High total protein diets have been linked to increased inflammatory bowel disease (IBD) risk [75]. Pea and whey protein consumption has been linked to increased levels of commensal *Bifidobacterium* and *Lactobacillus* [76], with fermented whey protein lowering counts of potentially pathogenic species *Bacteroides fragilis* and *Clostridium perfringens* [77]. Several genera that increase in abundance from ingestion of red meat are associated with higher levels of the proatherogenic chemical trimethylamine-N-oxide (TMAO), linked to an increase in cardiovascular disease risk [78]. High protein diets in individuals <65 years of age have been related to an increase in the production of insulin-like growth factor 1, a risk factor for cancers, diabetes, and mortality [79]. High fat diets in humans have been shown to increase the relative abundance of anaerobic bacteria and Bacteroidetes, while low fat diets increase fecal *Bifidobacterium* counts and reduce total cholesterol and fasting glucose [80]. In mice, there was a significant effect on microbiome composition when comparing polyunsaturated fat (fish oil) to saturated fat (lard) diets [81]. Lard-fed mice had increased systemic and adipose inflammation and lowered insulin sensitivity compared to the fish oil mice. Fecal transplantation replicated the metabolic and inflammatory phenotype, demonstrating a casual effect of microbiota [81]. The intestinal microbiome of animals fed on a high fat or high sugar diet has been observed to be more vulnerable to disruption of the circadian rhythm [82]. Given the particular importance of sleep to children, the combination of a poor diet and disrupted sleep may lead to alterations in gut microbiome composition.

### 4.2. Plant-Derived Prebiotics

Plant-derived non-digestible carbohydrates, such as some starches and fiber, survive in the colon, where they are fermented by the colonic microbiome to produce short chain fatty acids (SCFAs) [83]. Colonic epithelial cells utilize SCFAs, particularly butyrate, for 60–70% of their energy, and they contribute to strengthening of the mucosal barrier [84]. SCFAs also regulate glucose and lipid metabolism and immune function [73,85]. Butyrate acts as a histone deacetylase inhibitor, involved in the epigenetic control of regulatory T-cell production and maintenance [86]. Western diet-associated gut dysbiosis may promote a leaky gut membrane and metabolic endotoxemia [87], leading to increased cardiometabolic disease risk. A diet low in prebiotics decreases total bacterial load and diversity, while a high plant-based diet increases the gene richness of the microbiome and improves markers of inflammation [88,89,90]. In a study of commercially available infant cereal, different cereal types were associated with changes in microbiome composition, and differences in SCFA production in an in vitro infant gut model [91].

### 4.3. Other Dietary Modulators of Microbiome Composition

While the majority of diet–microbiome studies have focused broadly on Western vs. traditional diets, other dietary components have also been studied. Both undernourished and obese children in Mexico were found to have lower bacterial diversity compared with well-nourished normal-weight children [92]. There are limited data available on individual dietary effects on the microbiome in children, but evidence from adults suggest multiple diet-derived components shape microbiome composition. Gluten-free diets have been associated with changes in microbiota, with reductions in *Bifidobacterium*, *Lactobacillus*, and *Clostridium*, and increases in other bacteria including potential opportunistic pathogens [93,94]. Artificial sweeteners have been shown to modify gut microbiota [95] and have been suggested to induce glucose intolerance through their effects on gastrointestinal microbiota [96]. Other plant components, including polyphenols, may also modulate the gut microbiome [97,98,99]. Fermented foods act as a natural source of probiotics, with fermented dairy products [100,101] and vegetables [102,103] contributing bacteria to the diet, although the extent to which these bacteria survive and colonize the gut in individuals with established microbiomes is unclear [104]. Infant probiotic supplementation trials have generally found no long-term effects of supplementation on metabolic and inflammatory markers [105,106]. The microbiome adapts to available food sources; while some profiles may be more beneficial than others, there is a complex interaction between diet, microbiota, and downstream metabolic effects, which remains to be further studied.

## 5. Other Determinants of Microbiome Composition throughout Childhood

### 5.1. Effect of Antibiotics and Drug Interactions

Frequent use of antibiotics during childhood is associated with increased risk of antibiotic resistance [107] and may predispose individuals to increased risk of disease, including overweight and obesity [108,109], and inflammatory diseases [110], potentially through modulation of the microbiome. Use of antibiotics causes a decline in microbiome diversity and reduces resident beneficial commensals in the gut [111]. Low diversity is associated with increased body weight, insulin resistance, inflammatory tone, and dyslipidemia compared to higher gut diversity [112]. While broad-spectrum antibiotics have the greatest impact, the effects are mitigated, but still present, if the antibiotic is targeted to a specific pathogen. This disruption allows proliferation of both potentially pathogenic bacteria and those that promote obesity and metabolic dysregulation [113,114]. The impacts may be particularly large if these disruptions occur early in life, before the emergence of the relatively stable mature microbiome at two to three years old [115,116]. It takes several weeks for the microbiome to recover from a course of antibiotics, often never completely restoring to pre-antibiotic diversity [117]. Antibiotic use during the first 6 months of life was associated with higher risk of being overweight among children of normal weight mothers, although interestingly antibiotic use was associated with a decreased risk of being overweight among children of overweight mothers [118]. The association between increased body mass index (BMI) and early childhood cumulative exposure to antibiotics was found to be slightly higher if broad spectrum antibiotics were utilized [119]. A significant sex interaction has been reported, with antibiotic usage in the first 12 months of life associated with increased BMI in boys but not girls [120].

Emerging research is revealing interactions between the microbiome and drugs, both in how drug efficacy is modulated by microbiota and how use of pharmacological agents can shape the microbiome. Numerous drugs have been shown to be differentially metabolized by microbiota [121,122,123], and future consideration of microbiome composition could inform drug dosing in children. Various medications have also been shown to alter microbiome composition, including proton pump inhibitors, as well as statins and anti-diabetic drugs [124,125,126,127], although the long-term effects are unknown. Due to the important role the gut microbiome plays in the development of the immune system, it has been hypothesized that the gut flora may influence the host response to vaccines. While there have been relatively few studies investigating microbiome composition and vaccination, the data indicate that microbiota play a role in vaccine response [128]. A higher relative abundance of Actinobacteria and Firmicutes was associated with higher humoral and cellular vaccine responses, while high relative abundance of Proteobacteria and Bacteroidetes was associated with lower responses [129,130,131]. Whether vaccination induces any changes in the microbiome remains to be determined. Notwithstanding the life-saving benefits, data suggest that early and repeated use of antibiotics and potentially other medications during childhood is an important determinant of microbiome composition, which may impact future disease risk.

### 5.2. Sex and Genetic Differences in the Microbiome

Little is known about how host genetics may determine microbiome colonization during infancy, however, data in adults suggest that there is a genetic component to the microbiome [132,133,134]. Whether sex is an important modulator of microbiome composition in children remains unclear. Male infants were found to have a higher total bacterial count than female infants at birth, based on first defecation, while sex differences in *Lactobacillus* colonization were observed both at birth and after several months [135]. Sex differences in microbiome composition have been reported in adults [136], and these may interact with sex hormones to alter disease risk [137,138]. The microbiome may act as an intermediate mechanism linking genetics to disease, however, further research is required to understand potential causal links.

### 5.3. Environmental Modifiers of Microbiome Composition

Early exposure to pets has been associated with increased bacterial richness and diversity in the infant gut microbiome, which may protect against obesity and allergies [139,140]. The presence of siblings in the household has been associated with both increased diversity [141], as well as with reduced diversity and richness [139], and with differences in specific bacteria [111,135]. There is also evidence that exposure to urban and rural environments helps shape the microbiome in children [142]. Consistent with the hygiene hypothesis, it is likely that exposure to microbiota from pets and other children can contribute to development of a healthy microbiome. However, the relative importance of exposure within a shared household versus all other geographic and environmental exposures remains unclear.

### 5.4. Comorbidities

Significant medical events and comorbidities can have large effects on the microbiome. Hospitalization is associated with increased risk of *Clostridium difficile* colonization [111,143]. Changes in gut microbiota and increased translocation of intestinal bacteria have been found to occur following traumatic injury, including burns and traumatic brain injury [144,145]. While such injuries can by themselves cause long-term consequences in children, the effects of injury-related changes in microbiome composition are an important consideration that remain understudied. Autism has been associated with differences in microbiome composition [146], and treatments to alter microbiome composition including fecal transplant and probiotic supplementation have been shown to improve certain symptoms in children with autism [147,148]. Research is needed to clarify how comorbidities both drive, and are influenced by, differences in microbiome composition and function.

## 6. Mechanisms Linking Altered Microbiome Composition to Development of Obesity and Cardiometabolic Disease

As outlined, there are many different factors that shape the development of the microbiome, and many of these have also been linked to health outcomes. There is currently a knowledge gap, where we do not have prospective longitudinal or interventional studies that causally link altered microbiome composition to disease. However, as outlined, many studies suggest such a link, and fecal microbiome transplant studies have established causality in mice and to a limited extent also in humans [113,149,150,151,152]. At present, the specific mechanisms whereby the microbiome modifies cardiometabolic disease are less clear. Early events determining microbiome composition likely effect lifetime disease risk by altering long-term microbiome function. As expanded upon below, it is likely that microbiota affect cardiometabolic disease risk in the host through at least three potential mechanisms: (1) controlling nutrient bioavailability; (2) interacting with the immune system to modulate inflammation; and (3) production of specific protective or pathogenic metabolites. Evidence supporting these mechanisms is discussed below.

### 6.1. Energy Metabolism and Obesity

Numerous studies have identified differences in microbiome composition by body weight [153,154,155], with *Bifidobacterium* implicated in several studies linking the gut microbiome to obesity [52]. Fecal microbiome transplant experiments have shown that microbiota from obese humans or animals promote obesity, proving that microbiota themselves can cause obesity [113,150,156]. Further, fecal transplants have been shown to modulate insulin sensitivity in humans, although the long-term effects are unknown [151,152]. Simplistic calculations of the energy content of food and relationship to body weight assumes that the calories in a given food are equally available to all individuals. However, with an increased understanding of the role of microbiota, it is becoming clear that this is not the case [113,157]; because commensal microbes selectively metabolize food in the gut, they can control the supply of nutrients, including short-chain fatty acids to the host. Microbiota also contribute to biosynthesis of amino acids and vitamins, meaning the same foods may contribute different caloric and nutrient bioavailability to different people. Microbiota preferentially digest dietary prebiotics, including fiber, which are not easily digestible by the host. In individuals consuming low-fiber diets, insufficient supply of prebiotics may prompt changes in microbiome composition and metabolism that affects host metabolism [158]. While this process is complex, and confounded by many variables influencing body weight and food choice, subtle differences in energy availability over time could be enough to shift individuals from a lean to an obesogenic phenotype [159,160].

### 6.2. Inflammation

Inflammation is a key component of maintaining health, however, uncontrolled inflammation can have devastating consequences, with chronic low-grade inflammation promoting cardiometabolic disease development [161,162]. There is a complex interaction between commensal microbes and the host immune system, which, if dysregulated, can promote pathogenic inflammation. Although data suggest that the microbiome is partially modifiable throughout life, the initial development of the microbiome may have particular significance in establishing a core microbiome that is resistant to later modification. Dysregulation during early critical periods in infancy may have life-long implications on immune and metabolic function that may be difficult to reverse [163,164,165,166]. Experiments in mice have shown that age-dependent expression of TLR5 in the gut epithelium of neonates facilitates selective colonization by non-flagellated microbiota [166]. This suggests critical periods during development where exposure to specific bacteria allows normal development of host–microbiota relationships. Animal studies have shown that microbiota are required for normal intestinal development, and maturation of the immune system, through toll-like receptor 2 (TLR2)-mediated signaling [167,168,169]. In studies of delivery method and feeding in infants, differences in biomarkers of immune function including immunoglobulins and cytokines were observed concurrent with changes in the microbiome [12,170]. Translocation of microbes, microbial nucleic acids, and bacterial-derived lipopolysaccharide (LPS) from the intestine to the bloodstream occurs not only in the setting of intestinal disease but regularly in individuals with or without compromised gut barrier function, for example, during diet-induced post-prandial metabolic endotoxemia [87,112,171,172,173,174]. Bacteria and bacterial RNAs have been detected in tissues throughout the body, including atherosclerotic plaque and lipoproteins [175], suggesting that translocation of commensal microbes may play a causal role in disease etiology [176].

### 6.3. Modulation of Metabolites

Beyond effects on inflammatory signaling, microbiota also produce and modify other signaling molecules. Metabolites are generated in the gut by microbial metabolism, microbe–host interaction, and the action of microbiota on dietary substrates [4,177]. Gut microbial metabolism of dietary carnitine and choline to trimethylamine and subsequent hepatic oxidation to the pro-atherogenic metabolite TMAO was found to be associated with increased atherosclerotic risk [5,178,179], although whether TMAO is consistently pathogenic remains unclear [180]. Gut-mediated metabolism of soy food-derived isoflavones is associated with altered metabolism and cardiometabolic risk [181,182,183,184]. Many other diet–microbiome interactions may modulate disease risk through parallel mechanisms. The microbiome may also modulate disease biomarkers independent of dietary intake, including modulation of blood lipids [185], cardiovascular disease-related biomarkers [133], and glucose and insulin homeostasis [186].

## 7. Conclusions

As outlined throughout this review, there is considerable evidence linking gut microbiome composition to cardiometabolic disease pathophysiology. At present, our knowledge is mostly limited to observational studies and short-term interventions that permit some assessment of which factors affect gut microbiome composition and what consequences may be expected from dysregulation. However, large-scale prospective longitudinal studies are required to advance our knowledge of microbiome dynamics over time, and throughout disease development. While certain strategies to alter microbiome composition exist, including changes in diet or fecal transplant, at present these are non-specific and untargeted. Strategies that target specific bacteria or functional pathways may yield promising results but remain to be tested in appropriately-designed human trials. A major limitation is that we do not yet know what constitutes a “healthy” microbiome. Establishing this is imperative, but will be challenging, because a healthy microbiome is likely to be impacted by many factors and may differ between individuals and by life stage. Elucidation of the specific functions of different microbiota within the context of the host–microbial ecosystem is needed and may significantly impact our ability to understand the complex predictors of disease progression and identify targets for future microbiome therapeutics.
